# Causal Links Between Gut Microbiota and Vitamin Deficiencies: Evidence from Mendelian Randomization Analysis

**DOI:** 10.1007/s11596-025-00038-y

**Published:** 2025-04-07

**Authors:** Zi-xuan Hou, Wen-jing Li, Rong Pi, Han-wen-xi Wang, Meng-na Dai, Yan Ouyang, Su-yun Li

**Affiliations:** Department of Nursing, Union Hospital, Tongji Medical College, Huazhong University of Science and Technology, Wuhan, 430022 China

**Keywords:** Gut microbiota, Vitamin deficiencies, Mendelian randomization, Causal relationship, Genome-wide association study

## Abstract

**Objective:**

Vitamin deficiencies, particularly in vitamins A, B12, and D, are prevalent across populations and contribute significantly to a range of health issues. While these deficiencies are well documented, the underlying etiology remains complex. Recent studies suggest a close link between the gut microbiota and the synthesis, absorption, and metabolism of these vitamins. However, the specific causal relationships between the gut microbiota composition and vitamin deficiencies remain poorly understood. Identifying key bacterial species and understanding their role in vitamin metabolism could provide critical insights for targeted interventions.

**Methods:**

We conducted a two-sample Mendelian randomization (MR) study to assess the causal relationship between the gut microbiota and vitamin deficiencies (A, B12, D). The genome-wide association study data for vitamin deficiencies were sourced from the FinnGen biobank, and the gut microbiota data were from the MiBioGen consortium. MR analyses included inverse variance-weighted (IVW), MR‒Egger, weighted median, and weighted mode approaches. Sensitivity analyses and reverse causality assessments were performed to ensure robustness and validate the findings.

**Results:**

After FDR adjustment, vitamin B12 deficiency was associated with the class Verrucomicrobiae, order Verrucomicrobiales, family *Verrucomicrobiaceae*, and genus *Akkermansia*. Vitamin A deficiency was associated with the phylum Firmicutes and the genera *Fusicatenibacter* and *Ruminiclostridium 6*. Additional associations for vitamin B12 deficiency included the *Enterobacteriaceae* and *Rhodospirillaceae* and the genera *Coprococcus 2*, *Lactococcus*, and *Ruminococcaceae UCG002*. Vitamin D deficiency was associated with the genera *Allisonella*, *Eubacterium*, and *Tyzzerella 3*. *Lachnospiraceae* and *Lactococcus* were common risk factors for both B12 and D deficiency. Sensitivity analyses confirmed the robustness of the findings against heterogeneity and horizontal pleiotropy, and reverse MR tests indicated no evidence of reverse causality.

**Conclusions:**

Our findings reveal a possible causal relationship between specific gut microbiota characteristics and vitamin A, B12 and D deficiencies, providing a theoretical basis for addressing these nutritional deficiencies through the modulation of the gut microbiota in the future and laying the groundwork for related interventions.

**Supplementary Information:**

The online version contains supplementary material available at 10.1007/s11596-025-00038-y.

## Introduction

Vitamins are critical organic molecules necessary for human health, and trace amounts play key roles in physiological functions, growth, disease prevention, and treatment. Vitamin A deficiency is linked to clinical conditions such as xerophthalmia, nyctalopia, and increased vulnerability to severe infections. Deficiencies in vitamin B12 result in symptoms such as hyperpigmentation, vitiligo, jaundice, megaloblastic anaemia, and glossitis. Similarly, vitamin D deficiency is associated with a heightened risk of cardiovascular disease, Alzheimer’s disease, multiple sclerosis, and Parkinson’s disease. Mechanistic studies highlight that vitamins are indispensable for various physiological processes, including embryonic development, DNA synthesis, cell and immune system proliferation and differentiation, oxidative reactions, and bone metabolism. However, the etiology of vitamin deficiencies is multifactorial and includes inadequate dietary intake, medication use, and chronic gastrointestinal disorders.

Recent evidence underscores the pivotal role of the gut microbiota in vitamin synthesis and absorption, suggesting that imbalances in gut bacterial communities can directly impact vitamin metabolism. Research has demonstrated a correlation between alterations in the gut microbiota and vitamin deficiencies, with implications for conditions such as autism [1] and cholecystitis [2]. Vitamin A exists primarily as retinol or retinyl esters in animal products and as provitamin A carotenoids in plant foods. Gut bacteria are crucial for converting these forms into active retinoids, which are essential for vision, immune function, and cellular growth. Specifically, *Bacteroides* and *Clostridium* species influence the gut environment, enhancing the absorption of fat-soluble vitamins such as vitamin A, whereas *Lactobacillus* species promote the bioavailability and conversion of beta-carotene into active retinoids. Disruptions in the microbial balance of vitamin B12 can impair metabolic pathways involved in vitamin B12 absorption. Species such as *Akkermansia muciniphila* and members of the Bacteroides and Firmicutes phyla are implicated in vitamin B12 metabolism, possessing the enzymes necessary for its production and facilitating its absorption within the gut. Similarly, *Lactobacillus* and *Bifidobacterium* species are linked to vitamin D metabolism by increasing the bioavailability and conversion of vitamin D into its active forms. Despite extensive research highlighting the interaction between gut microbes and vitamins, the precise causal relationships between specific microbiota compositions and vitamin deficiencies remain poorly understood.

Mendelian randomization (MR) is a robust method that uses genetic variants as instrumental variables (IVs) to assess the causal relationship between exposures and outcomes, leveraging the random distribution of genetic variants during meiosis to minimize confounding. This approach provides more reliable causal inferences than observational studies do, mitigating residual confounding and reverse causality. In this study, we conducted a systematic two-sample MR analysis using single nucleotide polymorphisms (SNPs) identified from genome-wide association studies (GWASs) to explore the relationship between the gut microbiota and vitamin deficiencies. Genetic instruments associated with the gut microbiota were analyzed to determine their causal effect on vitamin deficiencies, aiming to clarify the potential role of the gut microbiome in influencing vitamin status.

## Materials and Methods

### Study Design

**Fig. 1:**
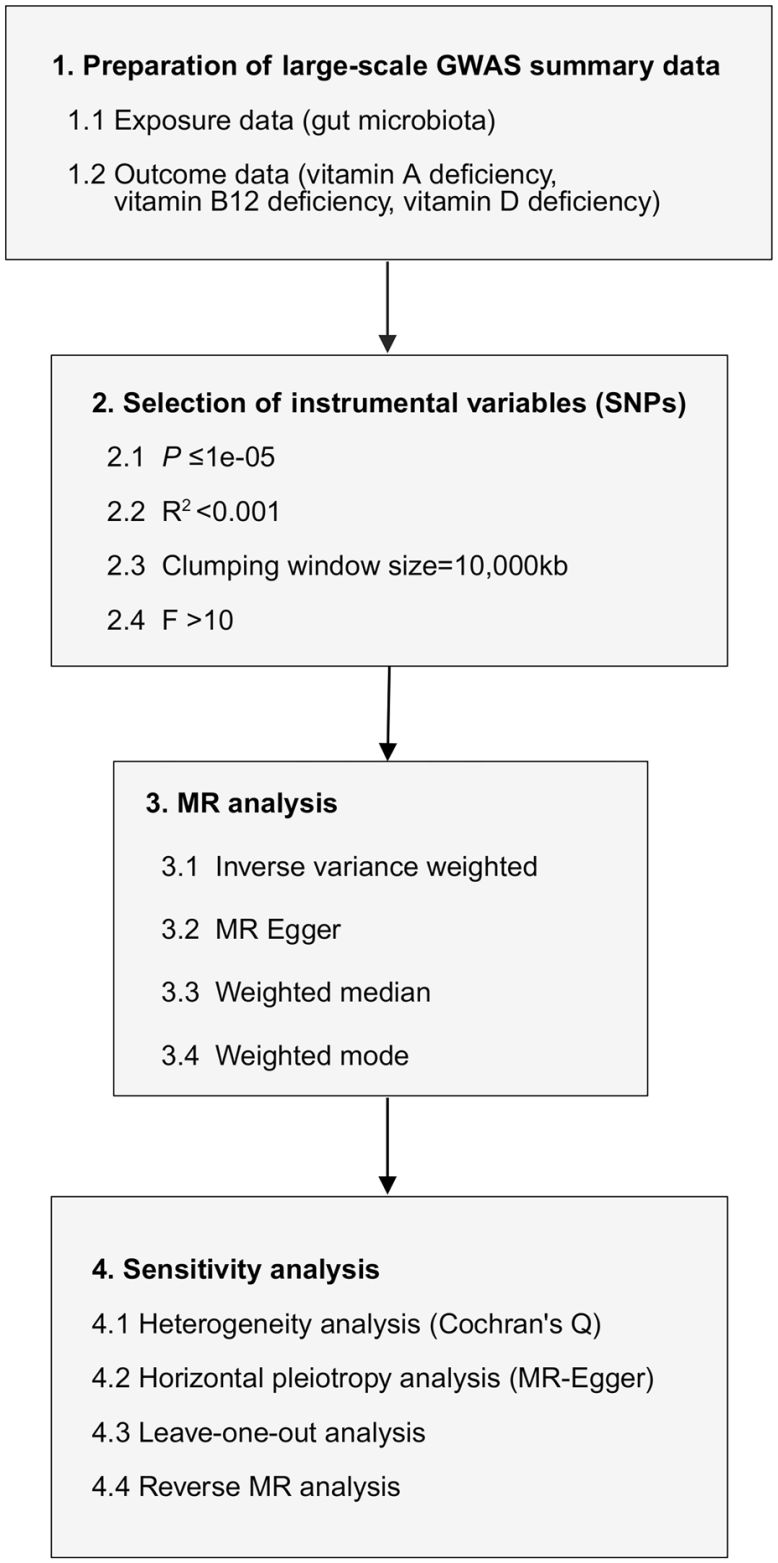
A schematic summary of the study design

The initial objective was to ascertain the causal relationship between the gut microbiota and vitamin deficiency. Upon confirming this relationship, we proceeded to investigate the causal links between the gut microbiota and deficiencies in vitamins A, B12, and D. A schematic overview of the study design is presented in Fig. 1. To reliably infer the potential causal relationship between the gut microbiota and vitamin deficiency via MR methods, it was necessary to satisfy three critical assumptions of MR analysis, including (1) IVs are associated with vitamin deficiency; (2) The IVs are unrelated to confounding factors that could influence this association; (3) IVs affect the gut microbiota solely through their impact on vitamin deficiency. The selection of data sources and instruments was described in the following section. A two-sample MR study was conducted using publicly available summary statistics from 199 GWASs: 196 for exposures and 3 for outcomes. To reduce the potential for population stratification bias, both the exposure and outcome cohorts were restricted to individuals of European ancestry. As the study used data sourced from publicly accessible databases, no further ethical approval or informed consent was necessary.

### Exposure Data

The MiBioGen consortium provides GWAS summary data for the gut microbiota and is currently the largest published source of human-related gut microbiota GWAS data. The study encompasses 24 cohorts, comprising 18,340 participants from a range of countries, including the United States, Canada, Denmark, and the United Kingdom. The majority of the participants were from Europe (*n* = 14,306). The GWAS data encompass 196 groups of bacteria classified into 5 taxonomic categories: class, phylum, order, family, and genus. The study focused on the genus level, identifying a total of 131 genera, with 12 unknown bacterial genera excluded from the subsequent analysis.

### Outcome Data

To obtain the exposure data, a search was conducted with the FinnGen biobank, which revealed no GWASs investigating deficiencies in other vitamins. Consequently, our attention was concentrated on three vitamins of particular interest: vitamin A, vitamin B12 and vitamin D deficiencies. The summary statistics for these deficiencies were obtained from the FinnGen biobank. The IVs were selected according to the following criteria: (1) we established a genome-wide significance threshold of *P* < 1 × 10^–5^, as this threshold has been shown to provide more comprehensive research results [3]; (2) to mitigate the impact of linkage disequilibrium and ensure that each SNP acts independently, we set *R*^2^ < 0.001 and clumping window size = 10,000 kb; (3) we excluded SNPs with an *F* statistic < 10 to reduce the risk of weak SNP bias; and (4) to ensure the completion of sensitivity analyses, we required a minimum of three SNPs to be included.

### Statistical Analysis

In the present study, two-sample MR was utilized to investigate the associations between the gut microbiota and deficiencies in vitamins A, B12, and D. The primary analysis was conducted via the IVW method, with additional analyses conducted via the MR‒Egger, weighted median, and weighted mode methods. Statistical significance was defined as a *P* value < 0.05 in the IVW method and consistent directions of effect across all four methods.

To assess potential pleiotropy, Cochran’s *Q* test for heterogeneity was applied, with a *P* value < 0.05 indicating significant heterogeneity. A “leave-one-out” analysis was also performed to evaluate the robustness of the IVW estimates and identify any influential SNPs. Horizontal pleiotropy was further examined via the MR‒Egger intercept term, with a *P* value < 0.05 indicating its presence. Additionally, a reverse MR approach and a global test were employed to investigate potential reverse causality between the gut microbiota and vitamin deficiency. To account for multiple comparisons, false discovery rate (FDR) adjustment was applied. All analyses were conducted via R software and the Mendel R package.

## Results

### SNP Selection

Following the exclusion of 15 taxa of unknown classification, our study included 196 taxa (9 phyla, 16 classes, 20 orders, 33 families, and 119 genera) for MR analysis. Following the application of rigorous IV selection criteria, 34 SNPs (2 for phylum, 2 for class, 3 for order, 6 for family, and 21 for genus) were identified at a significance level of *P* < 1 × 10^−5^. The *F* statistics for the selected SNPs associated with vitamin deficiencies (vitamins A, B12, and D) were all above 10, ranging from 17 to 36. This indicates a minimal risk of weak instrument bias. Accordingly, these SNPs were deemed suitable robust IVs with genome-wide significance. Further details regarding the selected IVs can be found in Table S1. The results demonstrate that all the SNPs functioned effectively as strong IVs, indicating minimal instrumental bias that could impact the MR analysis outcomes.

### Forward MR Analysis

**Fig. 2:**
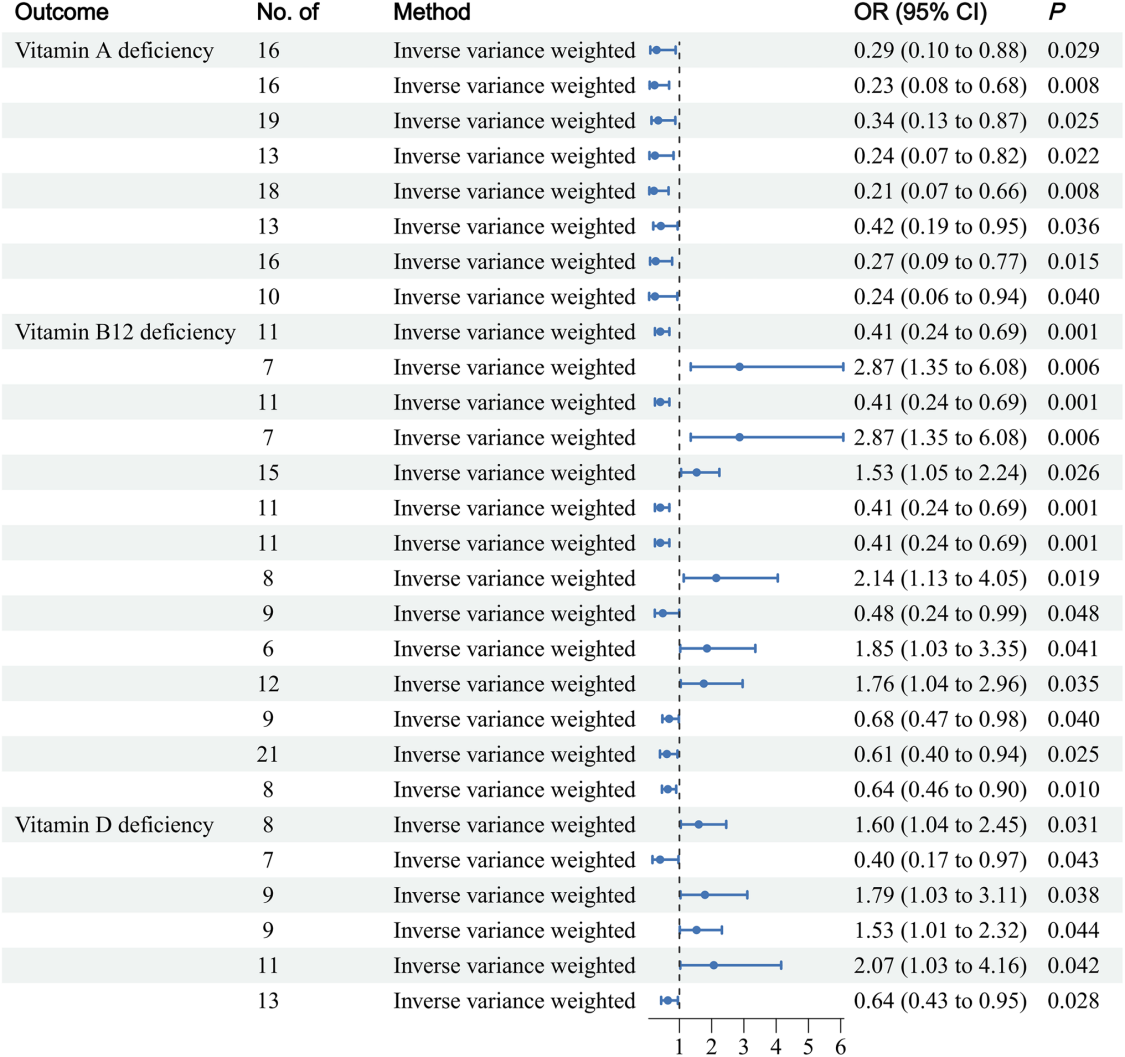
Mendelian randomization analysis of the gut microbiota taxa associated with different vitamin deficiencies

Following a series of adjustments and tests, we found that vitamin A deficiency was associated with the following taxonomic groups: the phylum Firmicutes (*P* = 0.029), the family *Prevotellaceae* (*P* = 0.008), the family Veillonellaceae (*P* = 0.025), the genus *Family VIII* (*P* = 0.022), the genus *Fusicatenibacter* (*P* = 0.008), the genus *Paraprevotella* (*P* = 0.036), the genus *Ruminiclostridium 6* (*P* = 0.015) and the genus *Ruminococcus 1* (*P* = 0.040). A deficiency in vitamin B12 was associated with the following taxonomic groups: class Verrucomicrobiae (*P* = 0.001), order Enterobacteriales (*P* = 0.006), order Verrucomicrobiales (*P* = 0.001), family *Enterobacteriaceae* (*P* = 0.006), family *Rhodospirillaceae* (*P* = 0.026), family *Verrucomicrobiaceae* (*P* = 0.001), genus *Akkermansia* (*P* = 0.001), genus *Coprococcus 2* (*P* = 0.019), genus *Coprococcus 3* (*P* = 0.048), genus *Enterorhabdus* (*P* = 0.041), genus *Lachnospiraceae* (*P* = 0.035), genus *Lactococcus* (*P* = 0.040), genus *Ruminococcaceae UCG002* (*P* = 0.025) and genus *Ruminococcaceae UCG011* (*P* = 0.010). A deficiency in vitamin D was associated with the following taxonomic groups: the genus *Allisonella* (*P* = 0.031), the genus *Eubacterium* (*P* = 0.043), the genus *Lachnospiraceae* (*P* = 0.038), the genus * Lactococcus* (*P* = 0.044), the genus *Ruminococcaceae UCG013* (*P* = 0.042) and the genus * Tyzzerella 3* (*P* = 0.028). Following adjustment for FDR (*P*_FDR_ < 0.05), 4 distinct gut microbiotas were identified as being significantly associated with vitamin B12 deficiency (*P* < 0.05). Among these genera, the class Verrucomicrobiae, the order Verrucomicrobiales, the family *Verrucomicrobiaceae* and the genus *Akkermansia* were identified as risk factors for vitamin B12 deficiency. However, we did not observe favorable outcomes for additional vitamin insufficiencies. Nevertheless, the results obtained are worthy of further consideration (Fig. 2).

Furthermore, the MR‒Egger analysis for vitamin A deficiency yielded results that were contrary to those of the IVW analysis, with the genera *Fusicatenibacter* and *Paraprevotella* identified as associated with this deficiency. Similarly, the genera *Coprococcus 3*, *Lactococcus*, and *Rhodospirillaceae* were associated with vitamin B12 deficiency. Additionally, the genera *Allisonella*, *Eubacterium*, and *Lactococcus* were associated with vitamin D deficiency. These results must be interpreted with caution. The remaining tests, including the MR‒Egger, weighted median, and weighted mode tests, corroborated the findings of the IVW analysis. Table S2 presents a comprehensive overview of the results obtained from the MR analysis.

**Fig. 3:**
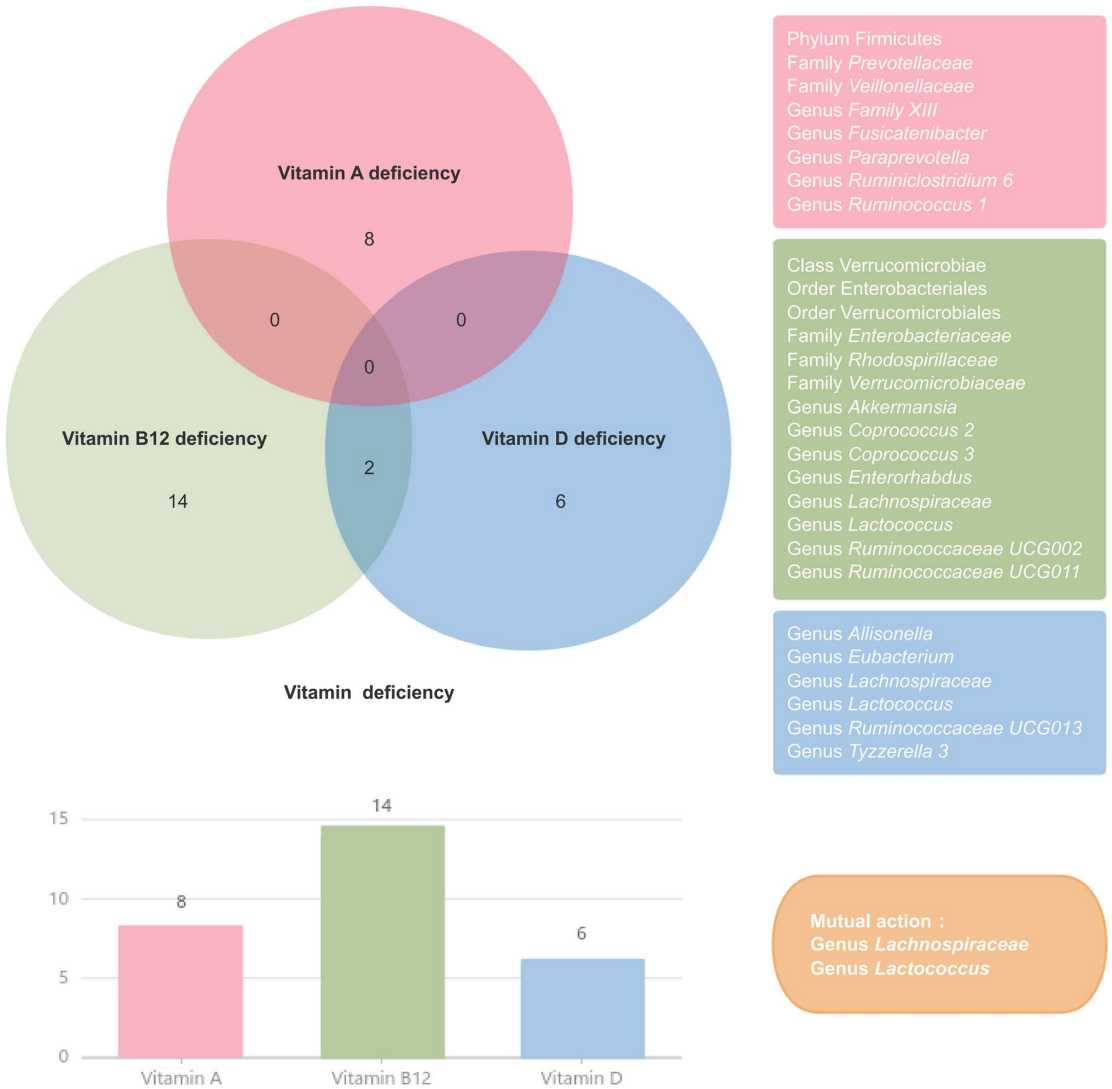
Venn diagram of the gut microbiota taxa associated with different vitamin deficiencies

Our research also revealed that multiple intestinal floras collaborate in the metabolism of various vitamins. Our study demonstrated that the genera *Lachnospiraceae* and *Lactococcus* interact in the processes of vitamin B12 and vitamin D deficiencies (Fig. 3). These findings indicate that the intestinal flora plays a pivotal role in the regulation of vitamin deficiency.

### Sensitivity Analyses

To ensure the reliability and robustness of our MR analysis results, we conducted heterogeneity and pleiotropy analyses. The Cochran’s *Q* statistic for IVW and MR‒Egger indicated the absence of heterogeneity in deficiencies of vitamins A, B12, and D. The MR‒Egger intercept and MR-PRESSO global test demonstrated the absence of potential horizontal pleiotropy, as detailed in Table 1. Additionally, the funnel plot provided further support for these findings (Figs. S1–S4). Furthermore, a leave-one-out sensitivity analysis was conducted, whereby each SNP was removed in turn. The results remained consistent throughout. The results of this analysis indicated that no single SNP was responsible for driving the causal association signal (Figs. S5–S8). For illustrative purposes, forest plots are provided in Figs. S9–S12, and scatter plots are shown in Figs. S13–S16, which facilitate the visualization of the data.

**Table 1:** Results of sensitivity analysis between gut microbiota and vitamin deficiency

Outcome	Exposure	Pleiotropy		Heterogeneity				Presso	
		MR–Egger		IVW		MR–Egger		Global test	
		Egger_intercept	*P*	Q	Q_pval	Q	Q_pval	RSSobs	*P*
Vitamin A deficiency	ebi-a-GCST90016948	0.122	0.386	12.507	0.640	11.707	0.630	14.101	0.684
	ebi-a-GCST90016956	0.029	0.704	8.590	0.969	8.441	0.956	10.016	0.966
	ebi-a-GCST90017008	0.041	0.875	12.593	0.399	12.564	0.323	15.404	0.383
	ebi-a-GCST90017011	–0.215	0.166	14.113	0.659	12.010	0.743	15.875	0.683
	ebi-a-GCST90017040	–0.146	0.392	10.260	0.593	9.467	0.579	11.949	0.603
	ebi-a-GCST90017050	0.057	0.648	7.515	0.942	7.298	0.923	8.579	0.945
	ebi-a-GCST90017062	–0.040	0.791	8.728	0.463	8.647	0.373	10.765	0.498
	ebi-a-GCST90017114	0.003	0.979	10.117	0.812	10.116	0.754	11.176	0.844
Vitamin B12 deficiency	ebi-a-GCST90016923	–0.053	0.470	5.958	0.819	5.389	0.799	7.137	0.854
	ebi-a-GCST90016936	–0.033	0.853	0.593	0.997	0.555	0.990	0.780	0.996
	ebi-a-GCST90016949	0.118	0.171	13.555	0.483	11.453	0.573	16.098	0.492
	ebi-a-GCST90016957	–0.053	0.472	5.952	0.819	5.387	0.799	7.129	0.847
	ebi-a-GCST90016961	–0.053	0.476	5.938	0.820	5.383	0.800	7.112	0.846
	ebi-a-GCST90016984	0.008	0.968	1.692	0.975	1.690	0.946	2.210	0.978
	ebi-a-GCST90016985	–0.136	0.288	4.204	0.838	2.884	0.896	5.307	0.850
	ebi-a-GCST90016992	0.033	0.768	2.559	0.768	2.459	0.652	3.412	0.804
	ebi-a-GCST90017021	–0.009	0.868	9.422	0.583	9.392	0.495	11.172	0.610
	ebi-a-GCST90017031	–0.146	0.220	8.380	0.397	6.568	0.475	10.258	0.453
	ebi-a-GCST90017053	0.017	0.704	21.377	0.375	21.212	0.325	24.299	0.357
	ebi-a-GCST90017059	0.126	0.311	4.252	0.750	3.025	0.806	5.338	0.794
	ebi-a-GCST90017098	–0.033	0.853	0.593	0.997	0.555	0.990	0.780	0.998
	ebi-a-GCST90017108	–0.053	0.470	5.958	0.819	5.389	0.799	7.137	0.851
Vitamin D deficiency	ebi-a-GCST90016963	0.167	0.471	9.318	0.231	8.482	0.205	12.901	0.235
	ebi-a-GCST90016998	–0.147	0.338	4.948	0.550	3.827	0.575	6.408	0.601
	ebi-a-GCST90017022	–0.076	0.598	9.500	0.302	9.103	0.245	12.170	0.321
	ebi-a-GCST90017031	0.226	0.114	7.210	0.514	3.954	0.785	9.582	0.515
	ebi-a-GCST90017060	–0.033	0.682	8.966	0.535	8.786	0.457	10.564	0.564
	ebi-a-GCST90017075	0.177	0.301	11.268	0.506	10.091	0.522	12.875	0.552

IVW, inverse variance weighted; RSSobs, residual sum of squares

### Reverse MR Analysis

The reverse MR analysis indicated that deficiencies in vitamins A, B12, and D were not causally associated with the gut microbiota, as demonstrated in Table 2. For a comprehensive overview of the results of the reverse MR, please refer to Table S3.

**Table 2:** Reverse MR analysis results between gut microbiota and vitamin deficiency

Outcome	Exposure		Method	Nsnp	β	SE	OR	OR_lci95	OR_uci95	*P*
Vitamin A deficiency	Phylum	Firmicutes	IVW	2	0.014	0.015	1.014	0.985	1.043	0.357
	Family	*Prevotellaceae*	IVW	2	–0.008	0.022	0.992	0.951	1.035	0.715
		*Veillonellaceae*	IVW	2	0.017	0.016	1.018	0.987	1.050	0.269
	Genus	*Family VIII*	IVW	2	–0.021	0.019	0.979	0.943	1.016	0.263
		*Fusicatenibacter*	IVW	2	–0.001	0.039	0.999	0.926	1.078	0.980
		*Paraprevotella*	IVW	2	–0.004	0.024	0.996	0.951	1.043	0.870
		*Ruminiclostridium 6*	IVW	2	–0.002	0.017	0.998	0.965	1.032	0.915
		*Ruminococcus 1*	IVW	2	–0.013	0.016	0.987	0.957	1.017	0.399
Vitamin B12 deficiency	Class	Verrucomicrobiae	IVW	6	–0.017	0.024	0.983	0.938	1.029	0.460
	Order	Enterobacteriales	IVW	6	–0.019	0.021	0.981	0.942	1.021	0.349
		Verrucomicrobiales	IVW	6	–0.017	0.024	0.983	0.938	1.029	0.460
	Family	*Enterobacteriaceae*	IVW	6	–0.019	0.021	0.981	0.942	1.021	0.349
		*Rhodospirillaceae*	IVW	6	0.010	0.031	1.010	0.951	1.072	0.751
		*Verrucomicrobiaceae*	IVW	6	–0.018	0.024	0.983	0.938	1.029	0.457
	Genus	*Akkermansia*	IVW	6	–0.017	0.023	0.983	0.939	1.029	0.460
		*Coprococcus 2*	IVW	6	–0.015	0.023	0.985	0.942	1.029	0.499
		*Coprococcus 3*	IVW	6	–0.005	0.019	0.995	0.958	1.033	0.788
		*Enterorhabdus*	IVW	6	–0.004	0.028	0.996	0.943	1.051	0.872
		*Lachnospiraceae*	IVW	6	–0.013	0.024	0.987	0.941	1.035	0.589
		*Lactococcus*	IVW	5	–0.016	0.046	0.984	0.899	1.078	0.733
		*Ruminococcaceae UCG002*	IVW	6	0.007	0.020	1.007	0.968	1.047	0.727
		*Ruminococcaceae* *UCG011*	IVW	5	–0.025	0.041	0.975	0.900	1.057	0.540
Vitamin D deficiency	Genus	*Allisonella*	IVW	6	0.021	0.034	1.021	0.955	1.092	0.542
		*Eubacterium*	IVW	8	–0.016	0.013	0.984	0.959	1.010	0.235
		*Lachnospiraceae*	IVW	8	–0.013	0.021	0.987	0.947	1.029	0.545
		*Lactococcus*	IVW	8	–0.047	0.033	0.954	0.894	1.018	0.157
		*Ruminococcaceae UCG013*	IVW	8	–0.005	0.013	0.995	0.970	1.021	0.715
		*Tyzzerella 3*	IVW	8	0.028	0.022	1.028	0.985	1.073	0.199

Nsnp, number of single nucleotide polymorphism; *β*, regression coefficient; SE, standard error; OR, odds ratio; OR_lci95, odds ratio lower confidence interval at 95%; OR_uci95, odds ratio upper confidence interval at 95%

## Discussions

Given the complex and poorly understood causal relationships between the gut microbiota composition and vitamin deficiencies, our study seeks to address these gaps by leveraging MR analysis. Recognizing the limitations of randomized controlled trials, MR provides a robust alternative by utilizing human genetic variations as IVs, which are independent of confounding factors. Specifically, this research investigated the potential causal links between deficiencies in vitamins A, B12, and D and the gut microbiota via large-scale GWAS summary statistics. Our findings suggest that gut microbiota contributes to vitamin deficiencies. These findings support a causal relationship and suggest microbiota-based interventions.

In the context of vitamin A deficiency, the two principal sources of vitamin A are retinol derived from meat and fish and the pro-vitamin A carotenoids present in fruits and vegetables. The intestinal absorption of retinol occurs in the lumen of the small intestine and is highly efficient, with an absorption efficiency between 70% and 90% [4]. Some studies suggest that vitamin A affects microbial composition. Conversely, other studies have suggested that sufficient vitamin A status may be linked to increased microbial diversity. The effects of vitamin A on mucus production may be responsible for these modulatory effects [5, 6]. Episodes of diarrhea are associated with an increased prevalence of vitamin A deficiency [7, 8]. Remodeling or altering the intestinal flora can lead to lower levels of bile acids, which reduces vitamin A absorption, increases RA levels by promoting vitamin A synthase and inhibiting vitamin A-degrading enzymes, and affects carotenoids, which affects vitamin A absorption [9]. Vitamin A is derived from retinoic acid in foods of animal origin or from a series of enzymatic reactions of carotenoids and carotenoids in plant foods [10]. Symbiotic gut communities can regulate vitamin A concentrations in coordination with the host via STRA6, a retinol-like transporter protein and activator of downstream signaling pathways [11]. Notably, consistent with previous studies, the abundance of specific bacterial genera, such as *Ruminococcus*, was negatively correlated with carotenoid levels, suggesting a complex interaction between the gut microbiota and vitamin A status [12], and to some extent, the effect of the gut microbiota on vitamin A deficiency may be mediated by carotenoid levels. Vitamin A also induces T-regulatory cells and inhibits IL-17 on mucosal surfaces to shift bacterial populations, resulting in a higher Firmicutes/Bacteroidetes ratio [13]. The impact of the gut microbiota on vitamin A has recently emerged as a topic of interest in the scientific community. This discovery has the potential to inform strategies for improving vitamin A deficiency by elucidating the pathways through which gut microbial communities influence this process.

Vitamin B12, which is uniquely synthesized by microorganisms, plays a critical role in human health, and the human gut microbiome is a significant contributor to its synthesis and utilization [14, 15]. Emerging research suggests that nearly 31% of the recommended daily allowance of vitamin B12 could be derived from the gut microbiota [16]. A previous study revealed a correlation between the gut microbiota and the occurrence of pregnancy-related anemia [17]. Nevertheless, the precise etiology of anemia remains uncertain. The present study offers a novel perspective on this matter, suggesting that alterations in the gut microbiota may contribute to the pathogenesis of anemia by modulating the pathway leading to vitamin B12 deficiency. The intestinal absorption of vitamin B12 is contingent upon the release of vitamins from food proteins, the normal secretion and function of intrinsic factors, and the appropriate gastrointestinal acidity [18]. An imbalance in the intestinal flora leading to an increase in anaerobic bacteria is thought to be an important factor in the symptoms of B12 deficiency [19]. In addition, the intestinal flora may influence vitamin B by modulating several other physiological factors, such as intestinal motility and acidity [20]. There is evidence that lactobacilli can synthesize vitamin B12, which may directly increase vitamin B12 levels in the body [21]. Moreover, probiotics may also have an indirect effect by improving gut health, particularly by maintaining the balance of the gut microbiota, which in turn enhances B vitamin absorption. On the other hand, B vitamins have the potential to increase the ability of probiotics to colonize the gut, directly increasing their bioavailability. In addition, B vitamins may indirectly enhance the efficacy of probiotics by positively influencing the gut microbiota [22]. This finding is consistent with our previous research, which highlighted the influence of gut microbiota on vitamin deficiencies. The findings of our study offer new insights that may inform the development of novel therapeutic strategies for the treatment of vitamin B12 deficiency. High-throughput genetic sequencing has also revealed the synergistic effect of B vitamins and probiotics in modulating the gut microbiota, particularly increasing the abundance of Verrucomicrobia and *Akkermansia* [22]. Vitamin B12 biosynthesis via selected human-related *Akkermansia* strains has been demonstrated [23], which is in agreement with our findings. Additionally, consistent with previous studies, *Ruminococcaceae* were more abundant in the low vitamin B12 group than in the high vitamin B12 group [24]. Although our study did not find reverse causality, they concluded that vitamin B12 intake may alter the total abundance of *Ruminococcaceae*.

Dietary vitamin D is efficiently absorbed in conjunction with dietary fat in the small intestine. An increasing body of clinical evidence supports the hypothesis that vitamin D has microbiome-modulatory effects [25]. Studies have demonstrated that microbiota and vitamin D profoundly influence each other and the immune system in many ways. Vitamin D plays a pivotal role as an intermediary between the immune system and the microbiota [26]. Recent studies have shown that vitamin D supplementation significantly increases gut microbial diversity, notably increasing the Bacteroidetes to Firmicutes ratio and promoting the growth of health-promoting probiotic taxa such as *Akkermansia* and *Bifidobacterium* [27]. Probiotics and pathogenic bacteria have also been shown to modulate vitamin D receptor expression, according to previous studies [28]. One study revealed a significant reduction in the abundance of *Blautia*, *Rosburia*, *Ruminococcus*, and *Dorea* after vitamin D supplementation [29], which are associated with increased gut permeability and inflammation [30]. The population at risk of vitamin D deficiency frequently includes individuals with inadequate sun exposure, limited oral intake, or impaired intestinal absorption [31]. Our research may provide new approaches for the treatment of vitamin D deficiency that involve not only the dose and concentration of the vitamin but also the absorption process in the gastrointestinal tract and the role of the gut microbiota.

Many gut microbes can absorb and transform vitamins, but they cannot produce them from scratch. These findings indicate that they have evolved to use vitamin co-factors produced by other organisms. Consequently, the biosynthetic capacity of the microbial community as a whole is crucial for individual species [16]. Bacteria not only synthesize vitamins for co-factors but can also recover intermediates and obtain vitamins from other species. The uptake and metabolism of vitamins by different bacterial species involve distinct metabolic pathways [32]. Furthermore, our research revealed that multiple intestinal floras collaborate in the metabolism of various vitamins, which indicates that the intestinal flora plays a pivotal role in the regulation of vitamin deficiency. These findings suggest that probiotics, prebiotics, or gut microbial transplantation could optimize vitamin absorption and metabolism, offering new strategies for managing vitamin deficiencies. However, further clinical trials are needed to confirm the efficacy of these interventions in improving vitamin deficiency symptoms.

Our study also has several limitations. First, the genome-wide statistical significance threshold (5 × 10^−8^) is too stringent to warrant further investigation; therefore, we included loci that met a relaxed significance level (1 × 10^−5^) in this study. Second, the GWAS summary data predominantly came from European populations, and the lack of gut microbiome data from other racial groups may introduce bias, limiting the generalizability of our findings. Further research is needed to assess the applicability of these results across diverse geographic and ethnic populations. Third, confounding factors such as diet, lifestyle, and medication use could influence both the gut microbiota and vitamin deficiencies, which may impact the interpretation of our findings. Finally, additional studies are necessary to investigate the underlying mechanisms connecting vitamin deficiency and the composition of the gut microbiota.

## Conclusion

The MR analysis identified 8 bacterial groups associated with vitamin A deficiency, 14 groups associated with vitamin B12 deficiency, and 6 groups associated with vitamin D deficiency. The reverse MR analysis demonstrated that deficiencies in vitamins A, B12 and D do not influence the gut microbiota. Notably, two bacterial groups were identified as commonly affecting vitamin deficiencies. Our research provides further corroboration and insights into the causal relationship between gut microbiota and vitamin deficiencies, offering avenues for the investigation of mechanisms and the identification of potential therapeutic targets. Further studies are needed to elucidate the biological mechanisms underlying the interaction between gut microbiota and vitamin deficiencies.

## Supplementary Information

Below is the link to the electronic supplementary materials. 

Supplementary Figs. S1–S4

Supplementary file6 (XLSX 101 kb)

## Data Availability

Publicly available datasets were analyzed in this study. These data can be found at: https://mibiogen.gcc.rug.nl/, https://www.finngen.fi/fi.
