# Supplementary material for: Causal Links Between Gut Microbiota and Vitamin Deficiencies: Evidence from Mendelian Randomization Analysis
Source: Curr Med Sci. 2025 Apr 7;45(2):321–30. doi: 10.1007/s11596-025-00038-y (PMC12053135; doi:10.1007/s11596-025-00038-y)
Supplement: Supplementary file 1 — Supplementary Figs. S1–S4 [file 11596_2025_38_MOESM1_ESM.pdf]

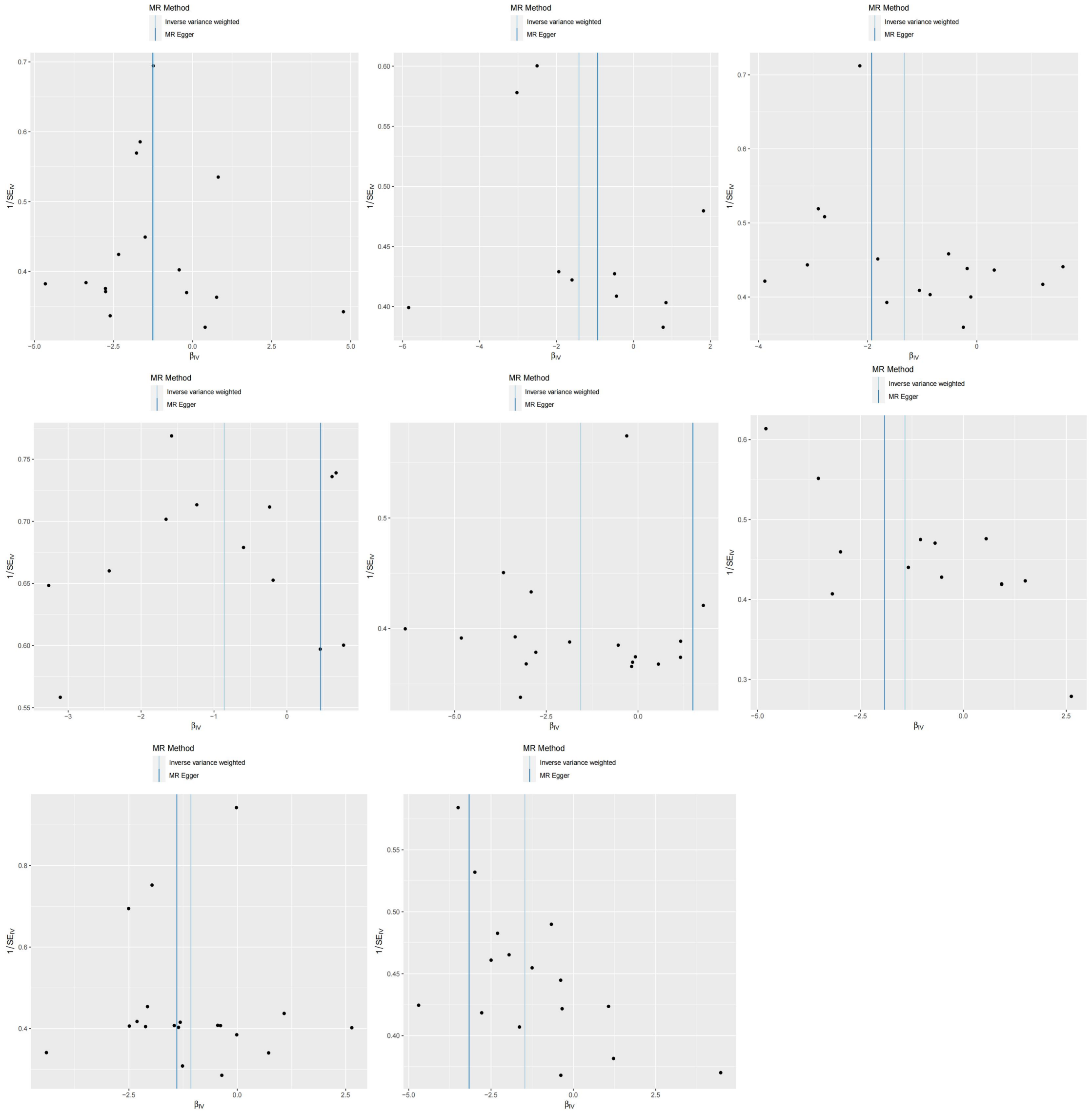

Figure S1. Funnel Plot of Vitamin A Deficiency

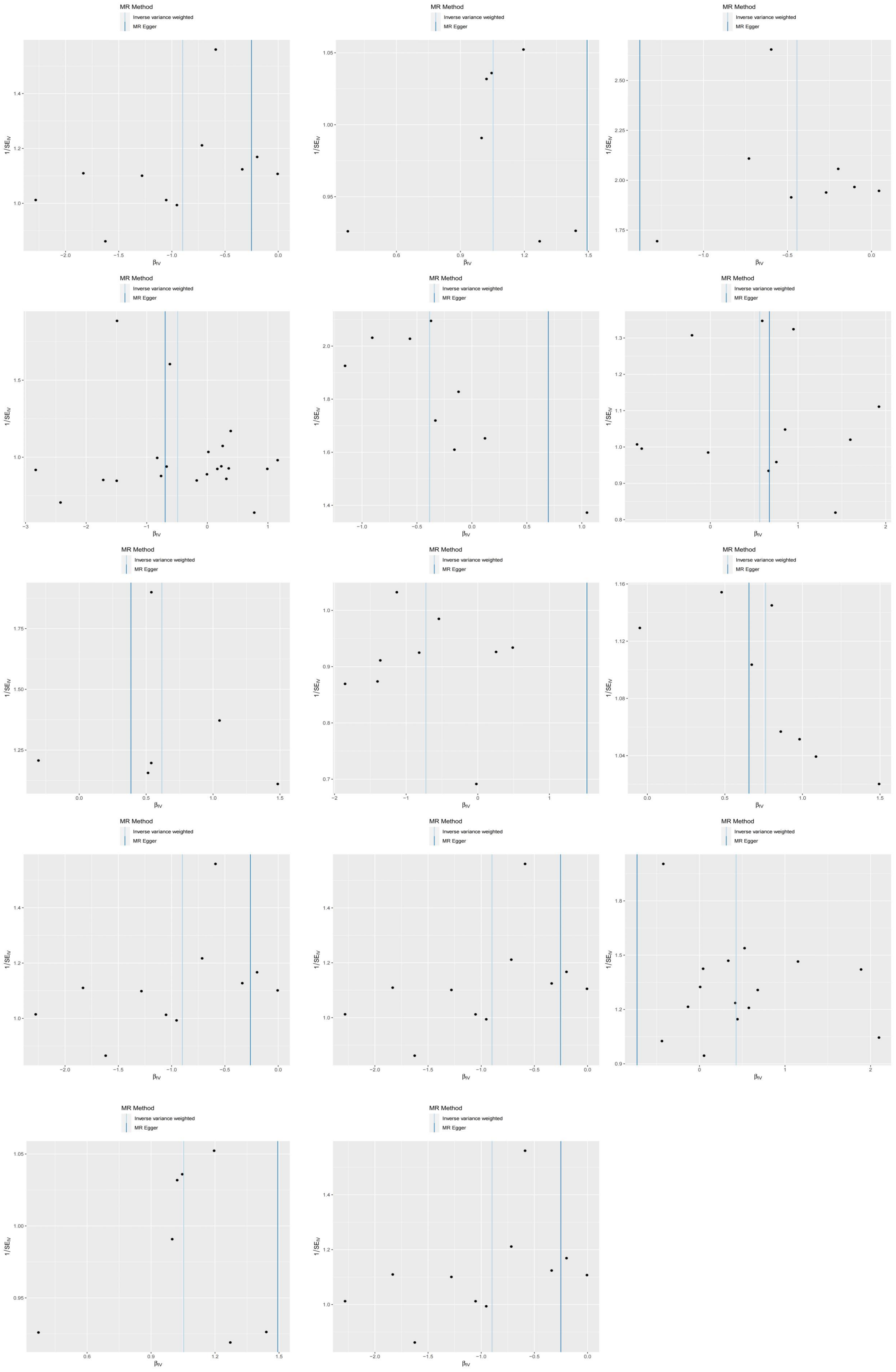

Figure S2. Funnel Plot of Vitamin B12 Deficiency

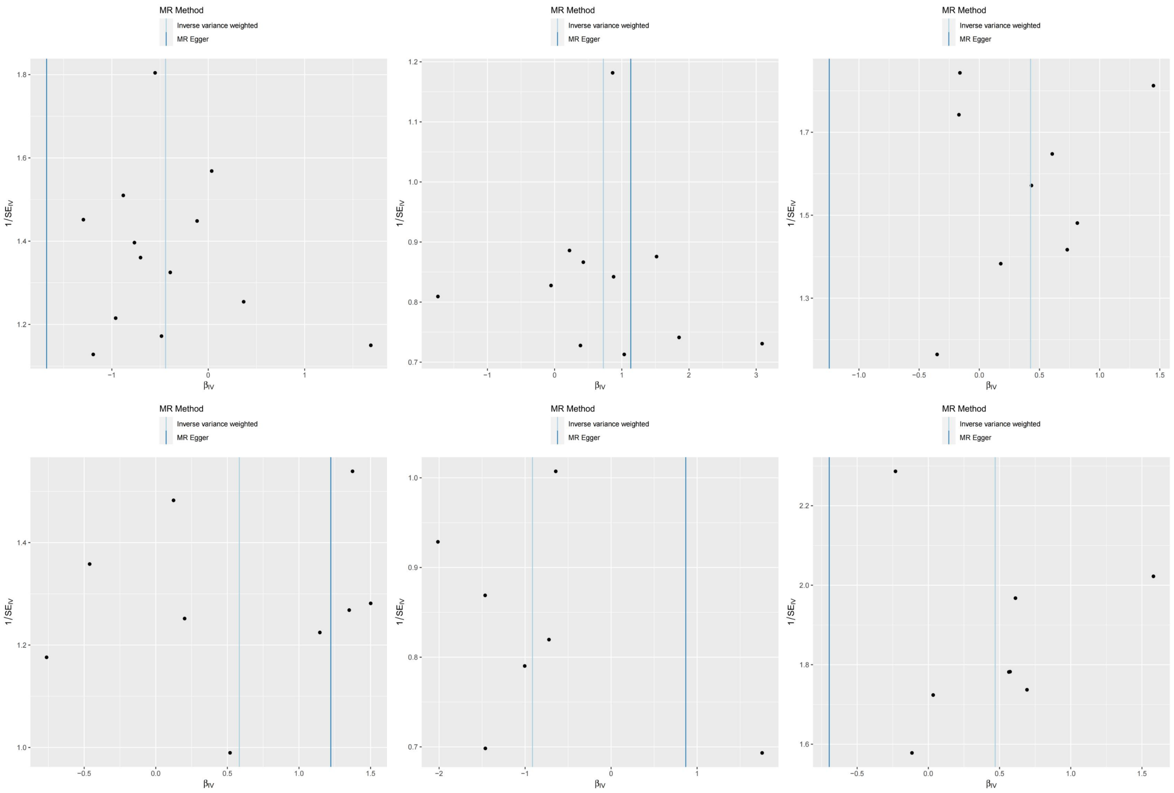

Figure S3. Funnel Plot of Vitamin D Deficiency

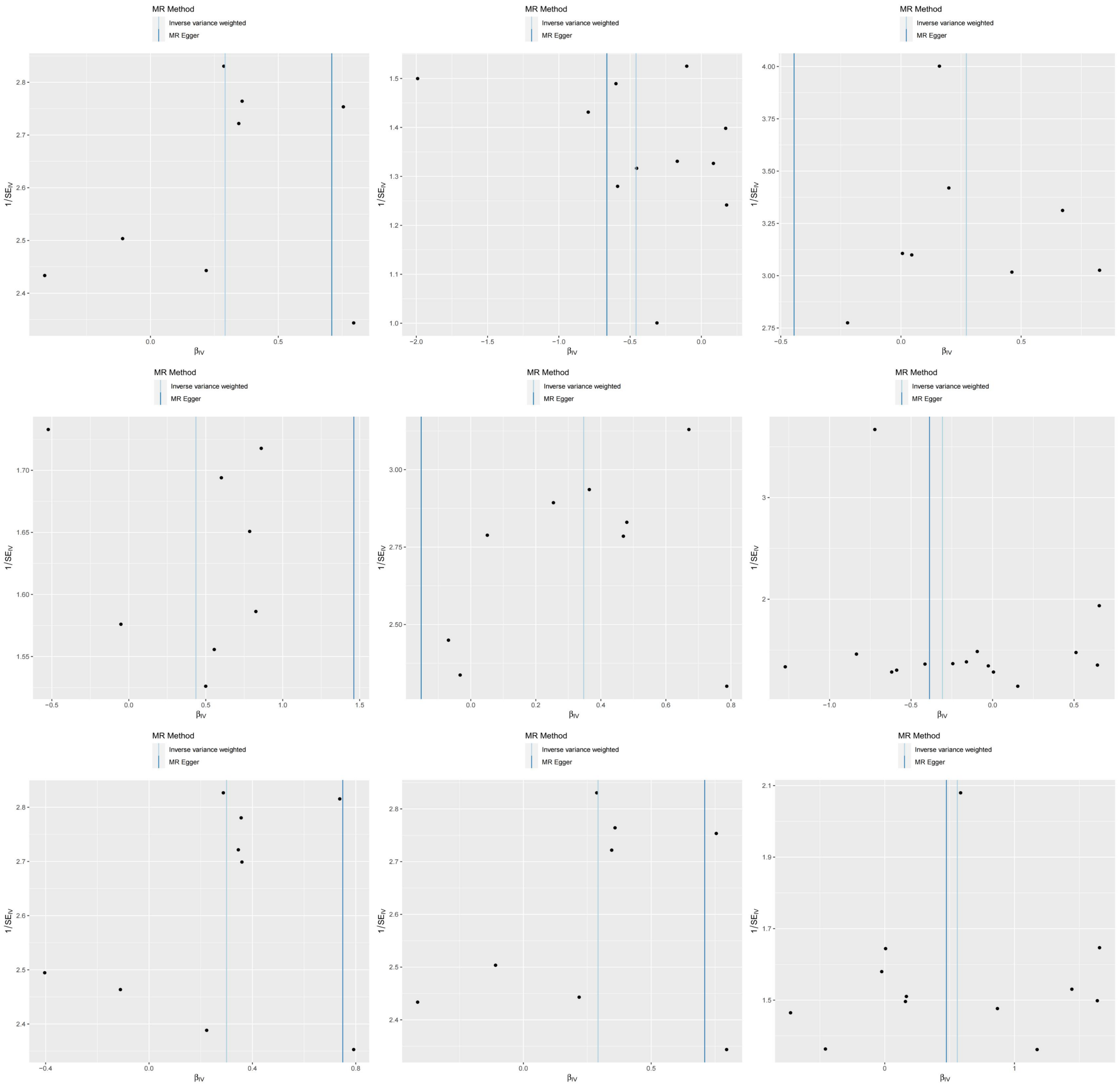

Figure S4. Funnel Plot of Vitamin Deficiency

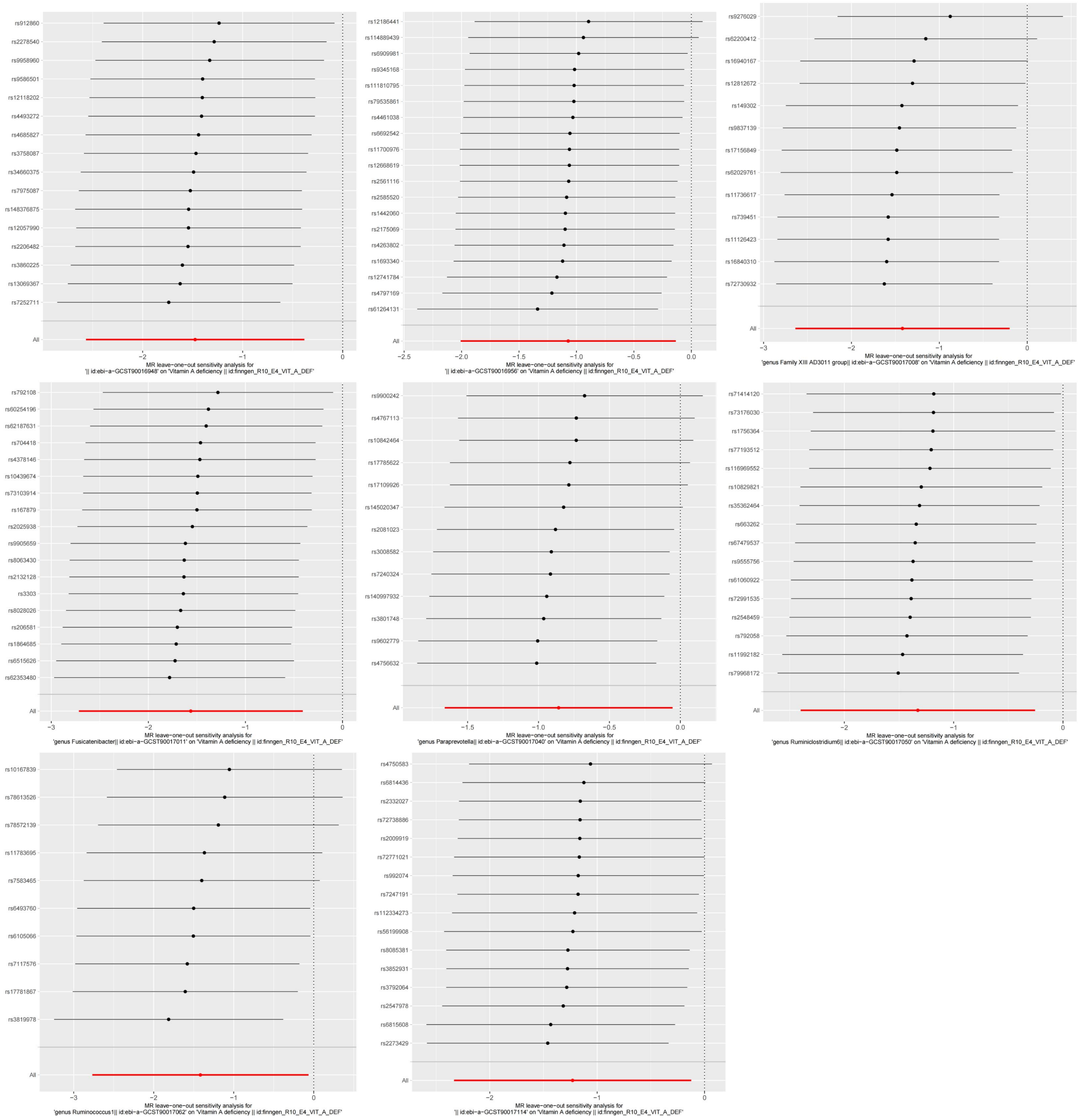

Figure S5. MR leave-one-out sensitivity analyes for Vitamin A deficiency

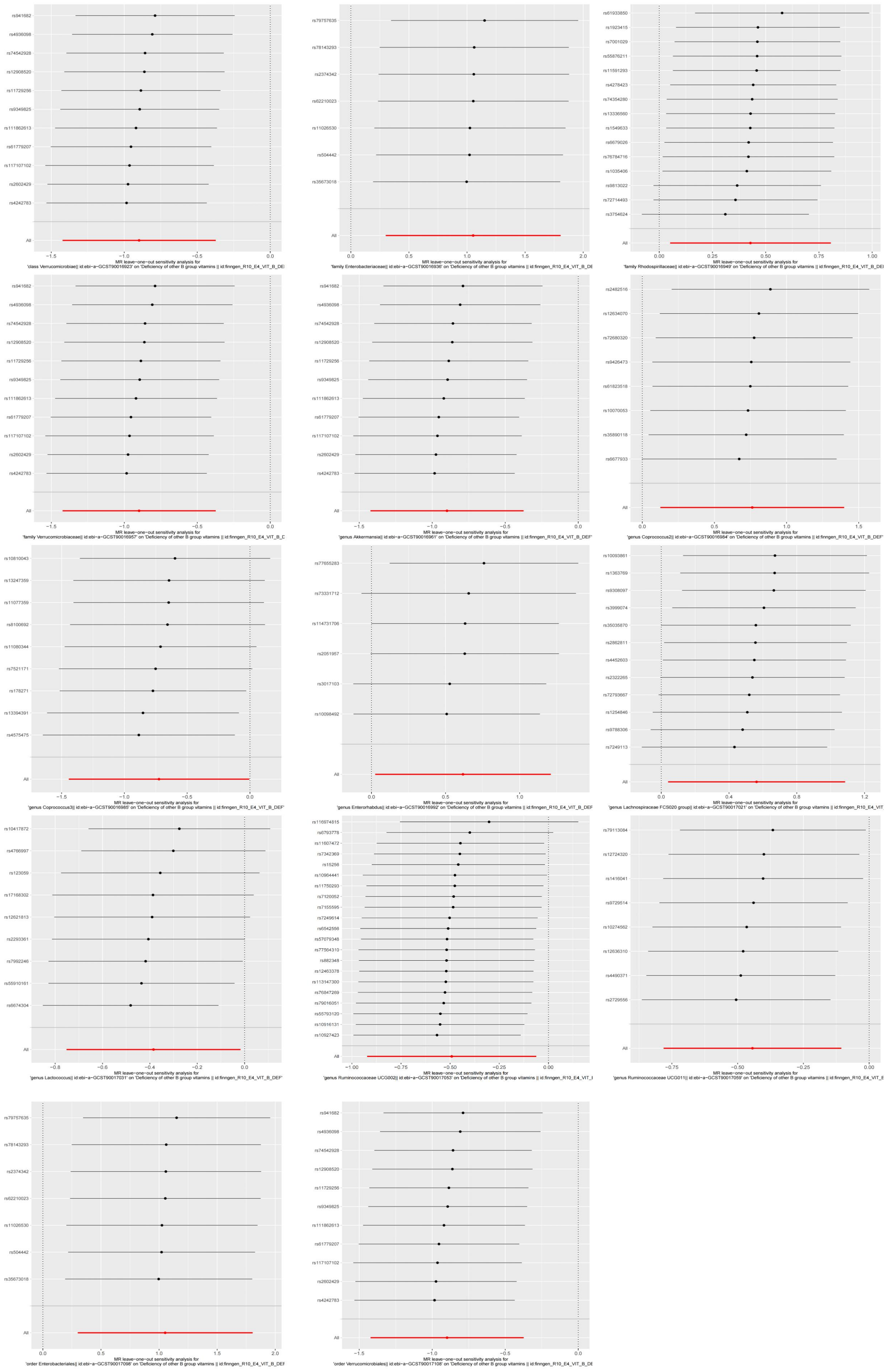

Figure S6. MR leave-one-out sensitivity analyes for Vitamin B12 deficiency

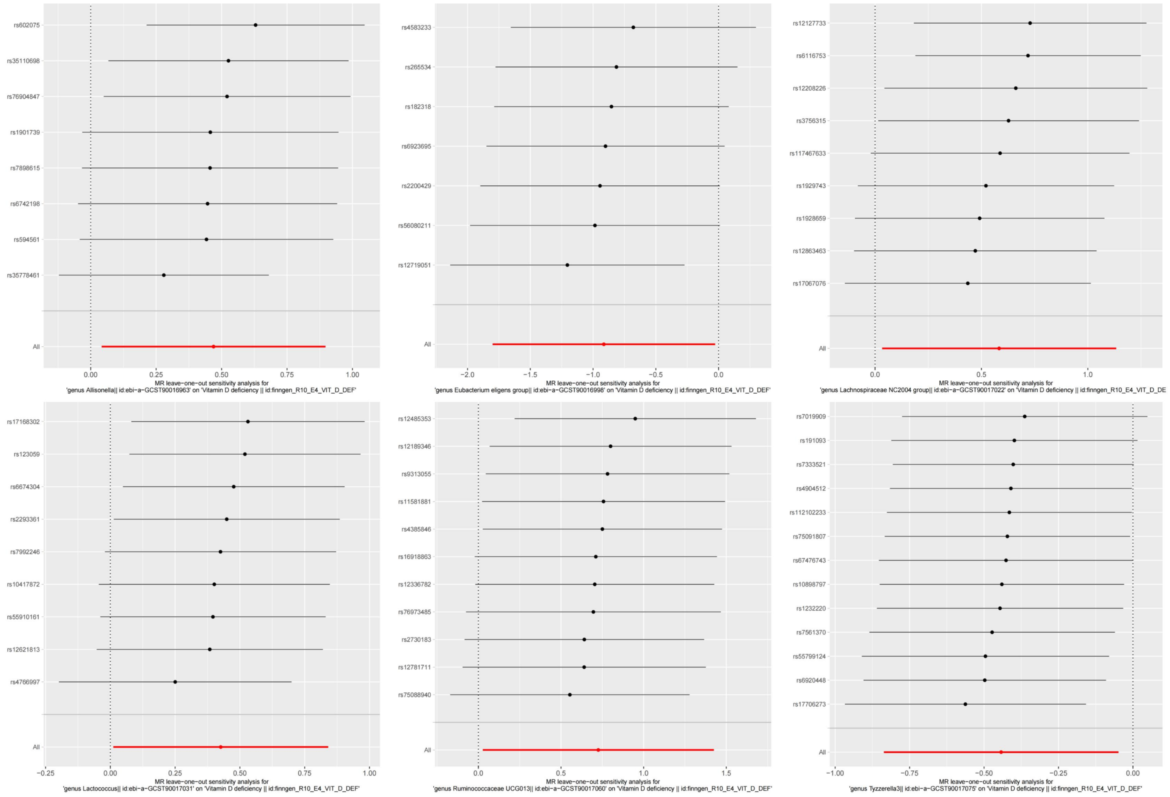

Figure S7. MR leave-one-out sensitivity analyes for Vitamin D deficiency

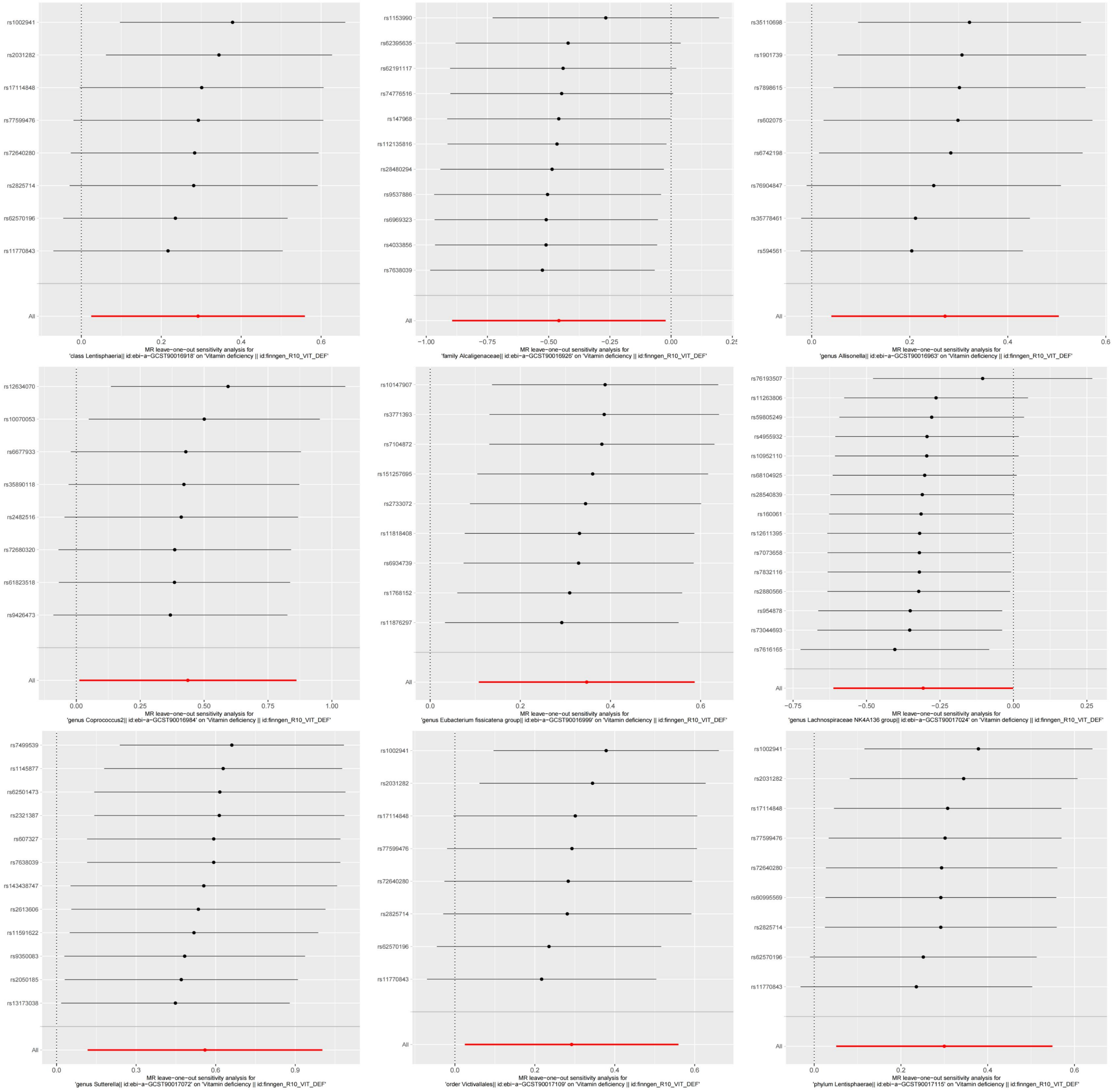

Figure S8. MR leave-one-out sensitivity analyes for Vitamin deficiency

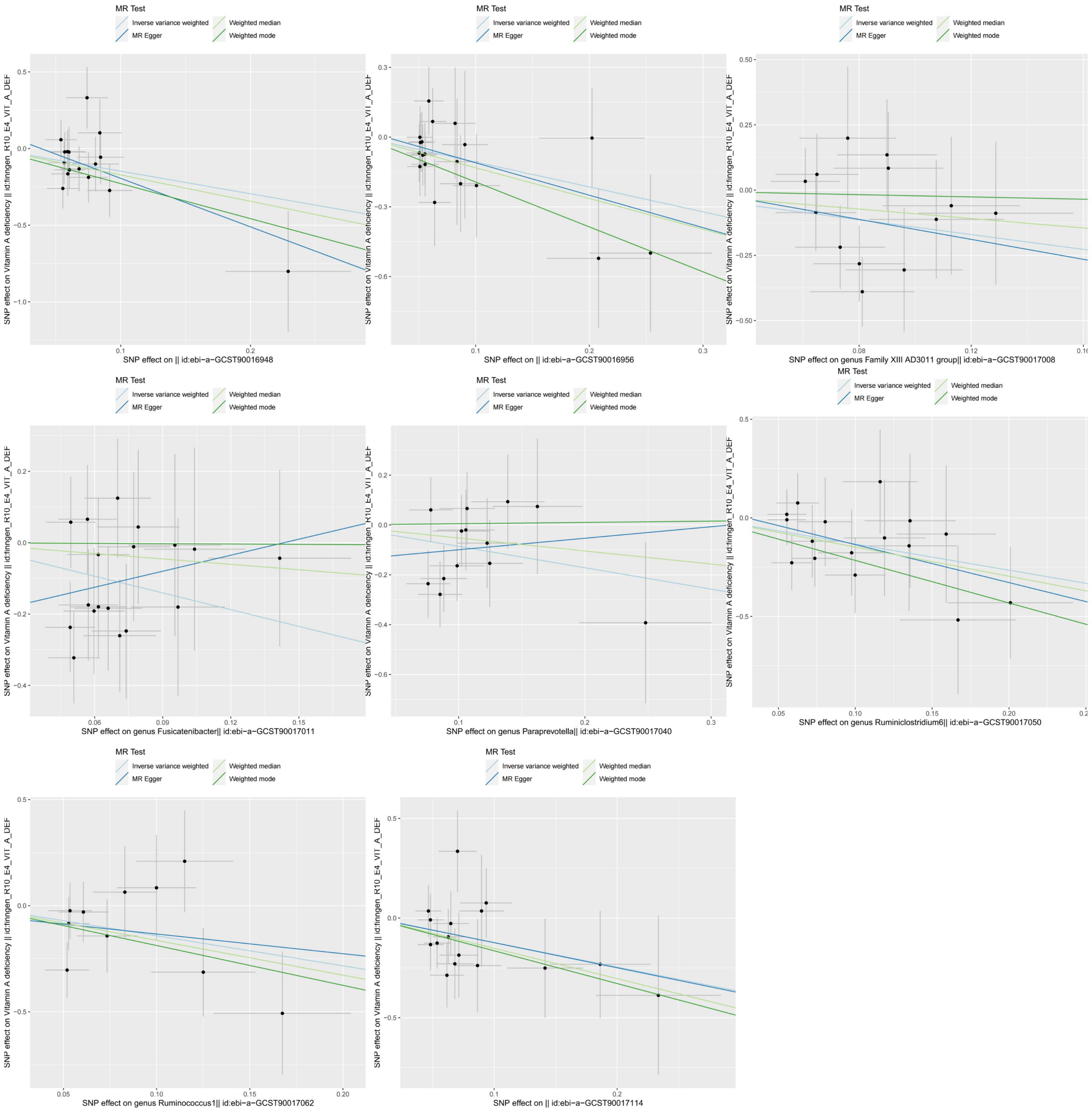

Figure S9. Forest Plot of Vitamin A Deficiency

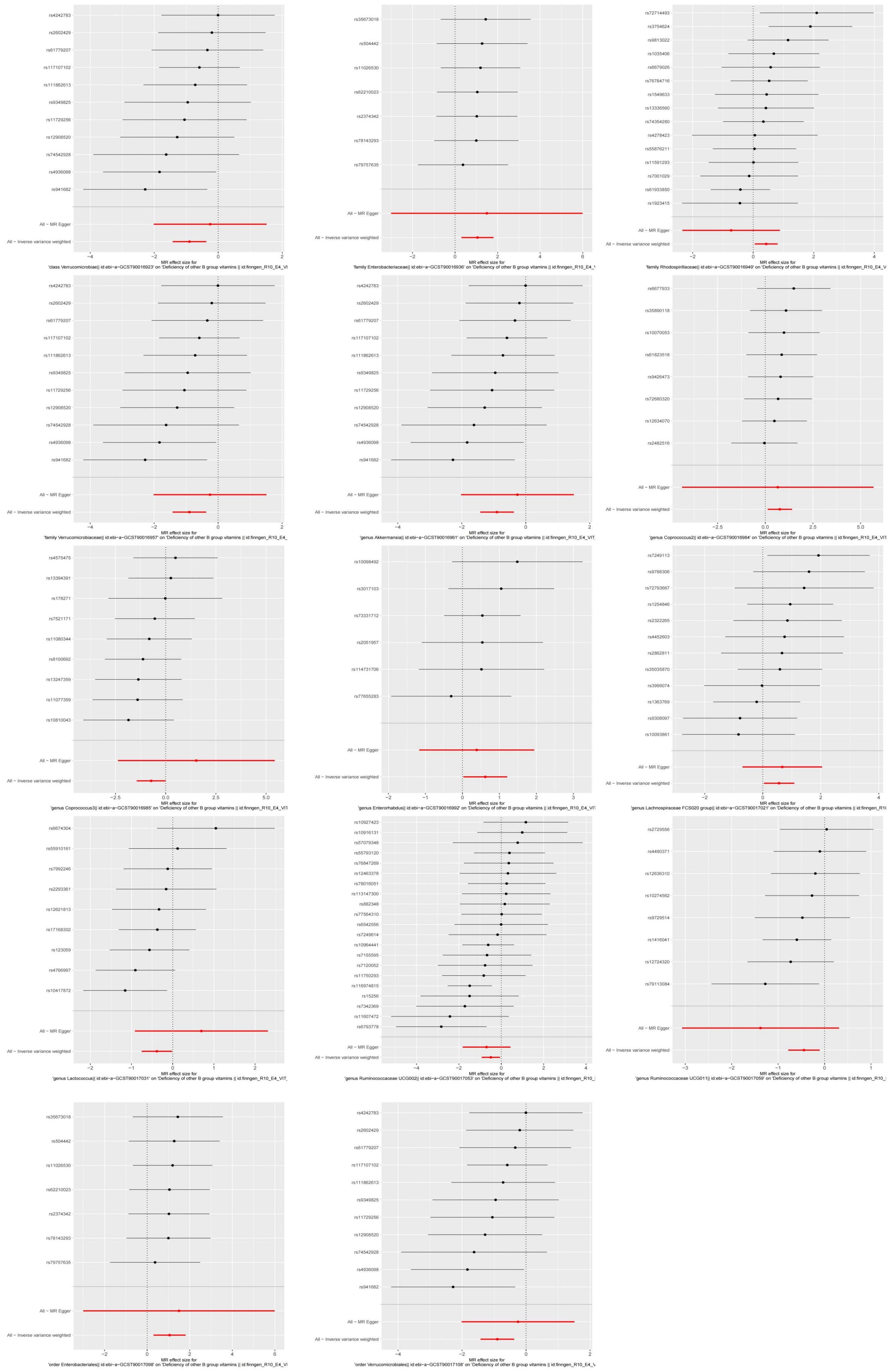

Figure S10. Forest Plot of Vitamin B12 Deficiency

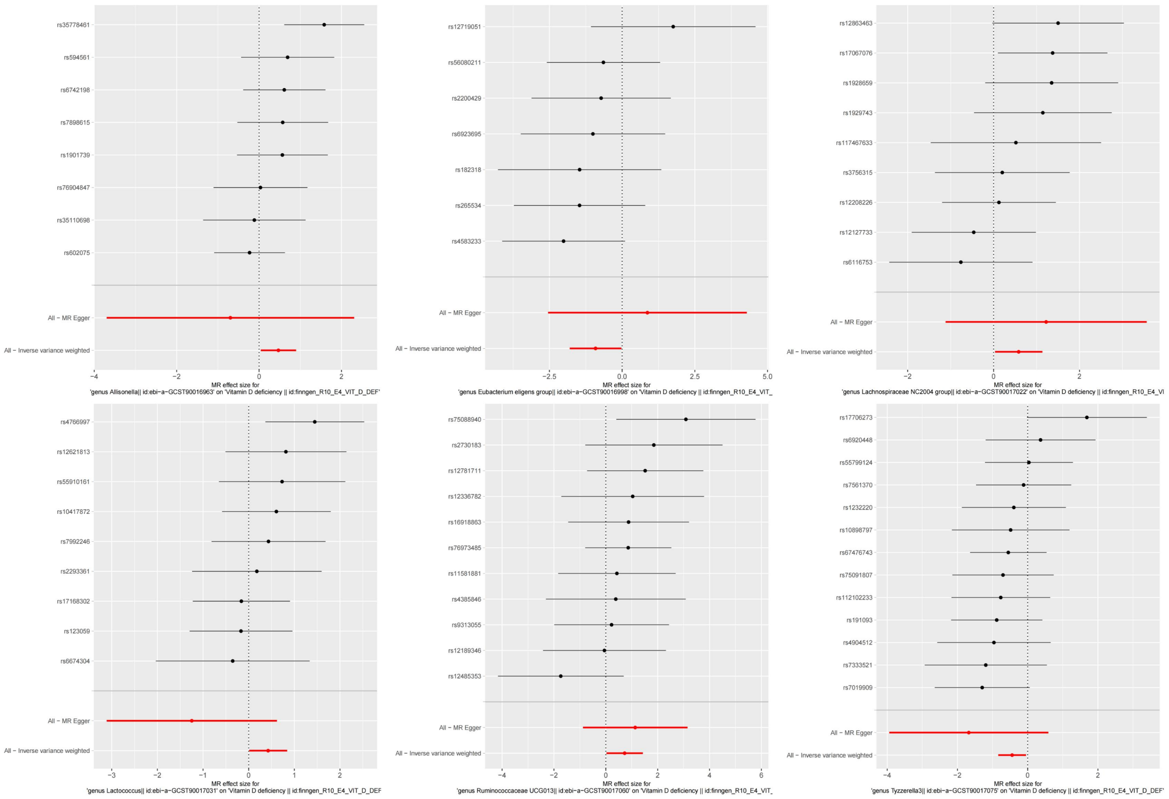

Figure S11. Forest Plot of Vitamin D Deficiency

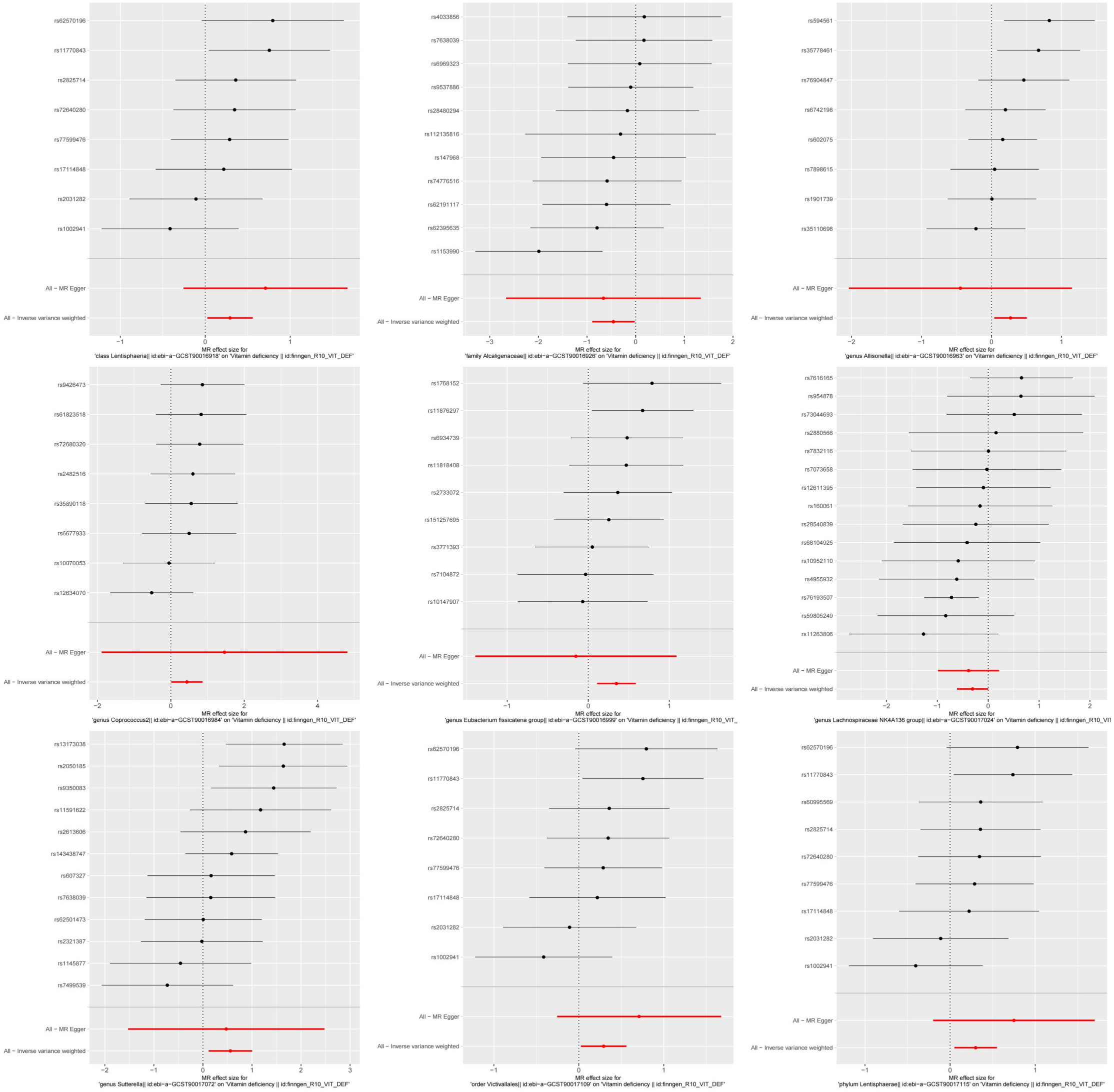

Figure S12. Forest Plot of Vitamin Deficiency

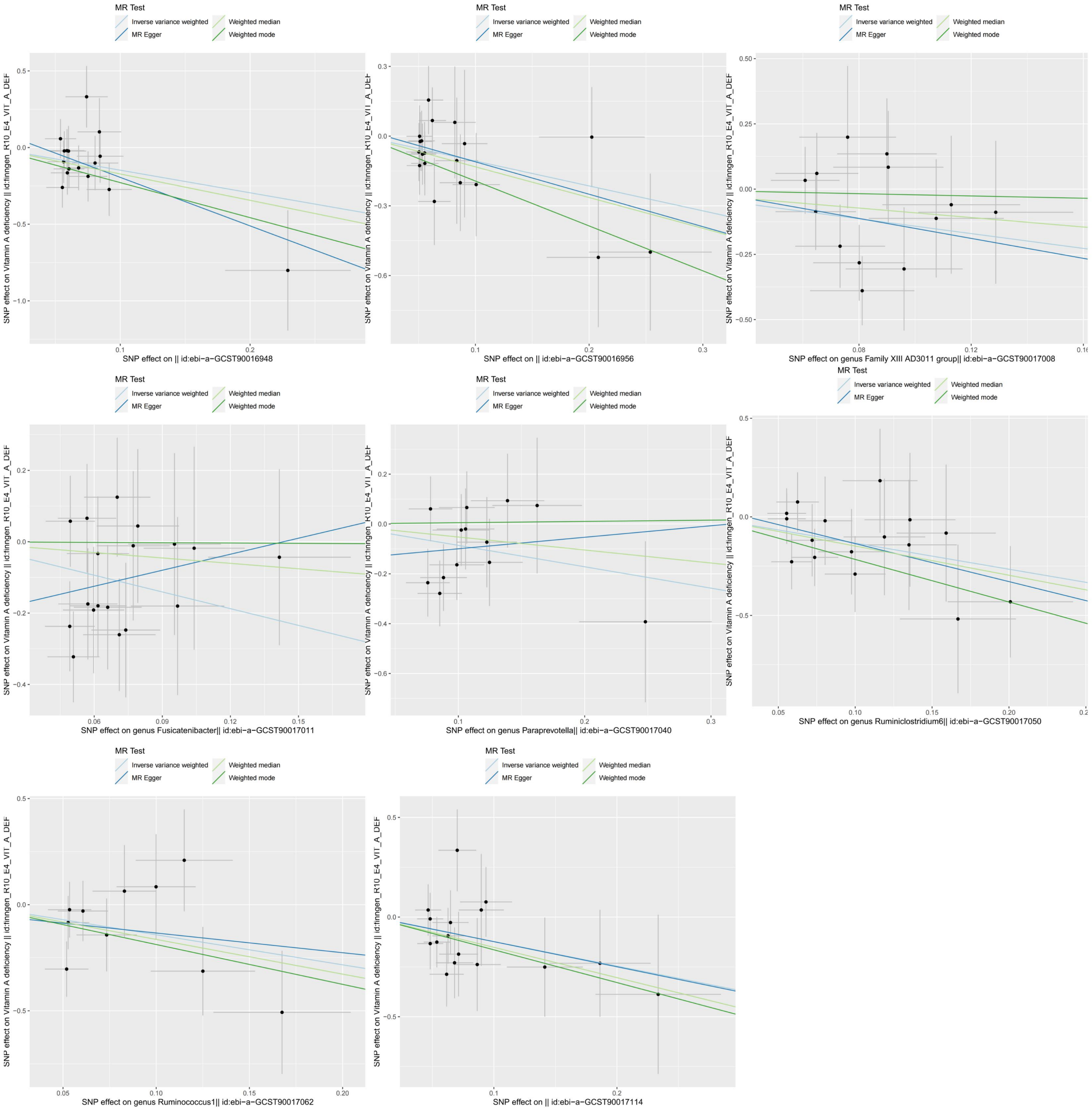

Figure S13. Scatter Plot of Vitamin A Deficiency

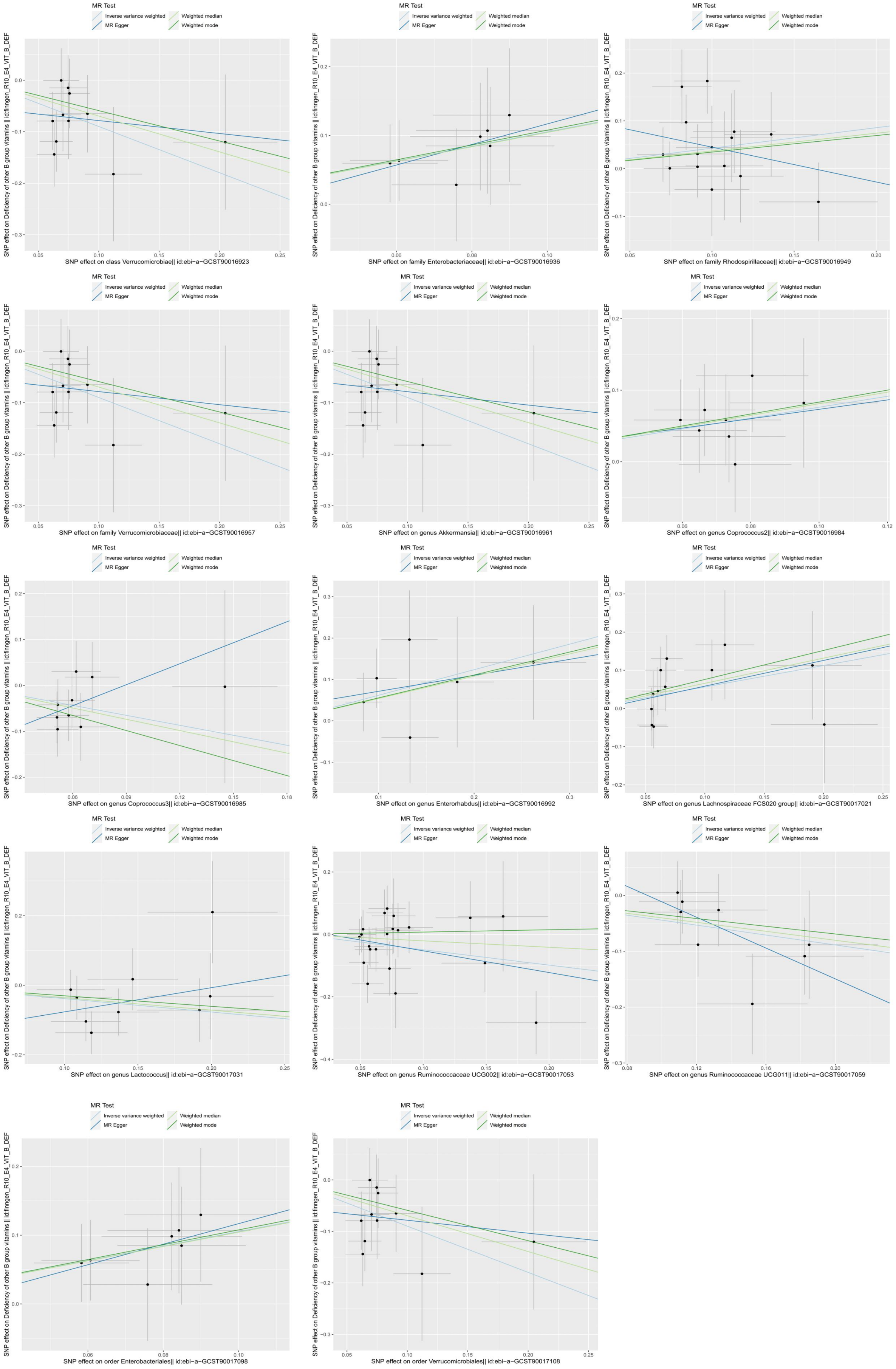

Figure S14. Scatter Plot of Vitamin B12 Deficiency

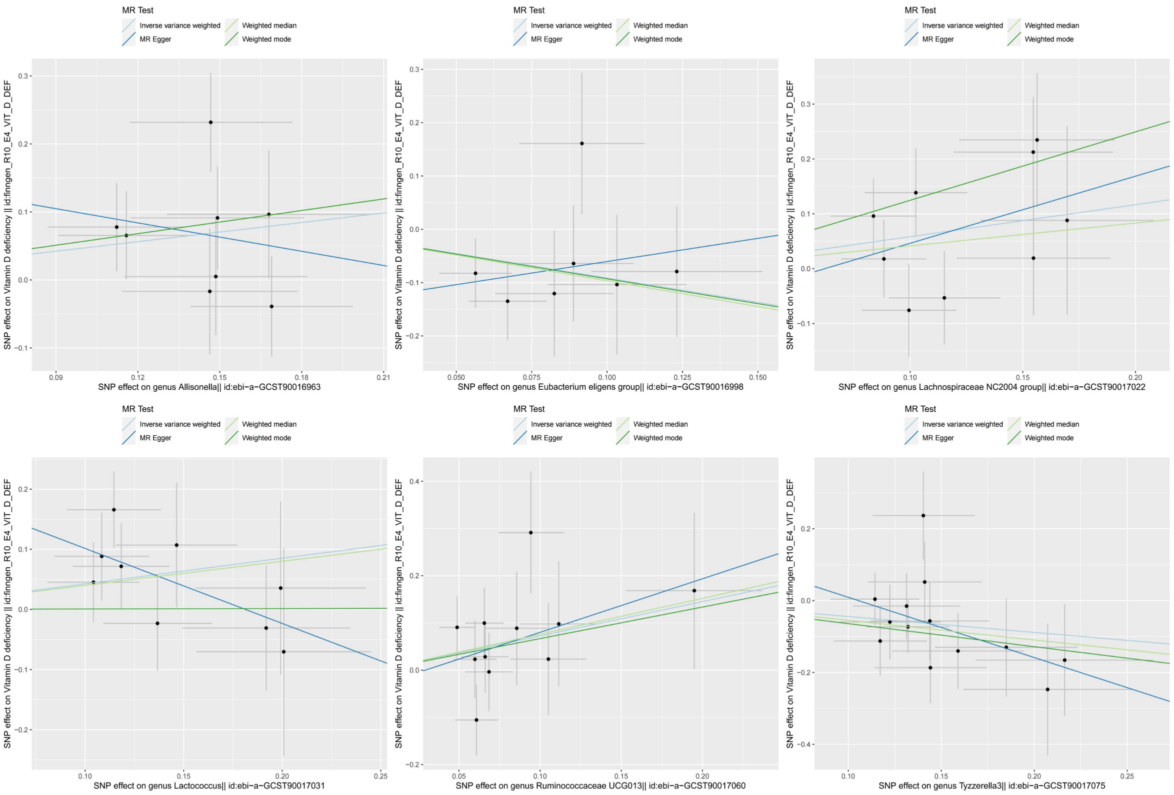

Figure S15. Scatter Plot of Vitamin D Deficiency

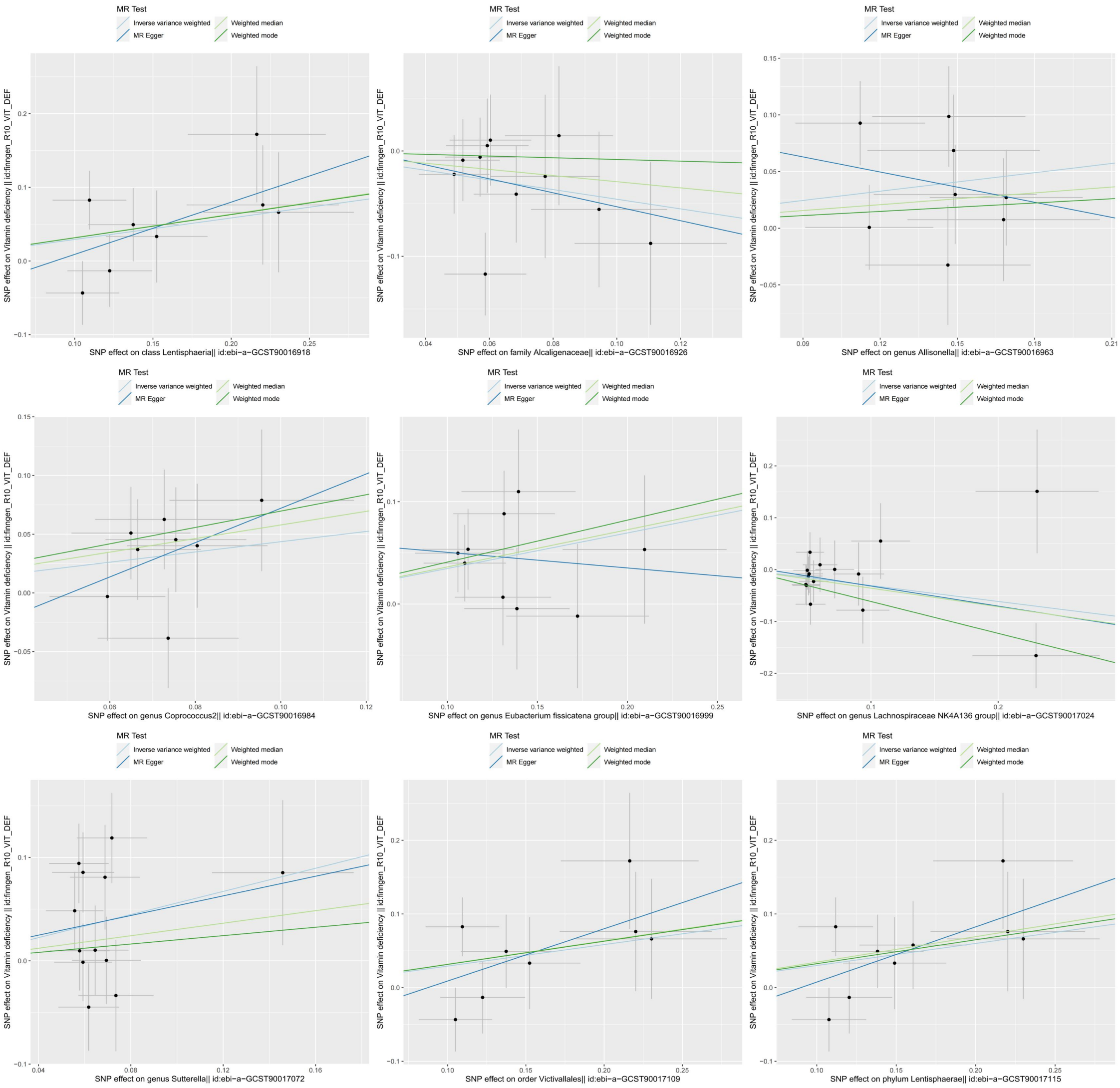

Figure S16. Scatter Plot of Vitamin Deficiency
